# MiR-204-5p regulates SIRT1 to promote the endoplasmic reticulum stress-induced apoptosis of inner ear cells in C57BL/6 mice with hearing loss

**DOI:** 10.1371/journal.pone.0309892

**Published:** 2024-11-12

**Authors:** Yaqin Hu, Xiaoqin Luo, Hongjiang Chen, Jing Ke, Menglong Feng, Wei Yuan

**Affiliations:** 1 Chongqing Medical University, Chongqing, China; 2 Department of Otolaryngology, Chongqing General Hospital, Chongqing, China; 3 Hospital of Traditional Chinese Medicine Affiliated to Southwest Medical University, Luzhou, China; University of Michigan, UNITED STATES OF AMERICA

## Abstract

**Purpose:**

This study investigated the effect of miR-204-5p-mediated silencing of SIRT1 on the development of deafness in C57BL/6 mice and the roles of miR-204-5p and SIRT1 in deafness.

**Methods:**

Auditory brainstem response recordings, H&E staining, and immunohistochemistry were used to observe changes in hearing function and cochlear tissue morphology in 2-month-old and 15-month-old C57BL/6 mice. A senescence model was induced using H_2_O_2_ in inner ear cells (HEI-OC1). Changes in HEI-OC1 cell proliferation were detected using the CCK-8 assay, whereas flow cytometry was used to detect changes in apoptosis. MiR-204-5p expression was measured via RT‒qPCR. The SIRT1 agonist RSV and a miR-204-5p inhibitor were used to study changes in ER stress (ERS), proliferation, and apoptosis in HEI-OC1 cells. Western blotting was performed to detect changes in ATF4, CHOP, SIRT1, PERK, p-PERK, Bax, and Bcl-2 protein levels. A dual-luciferase reporter gene assay was carried out to assess the ability of miR-204-5p to target SIRT1.

**Results:**

Relative miR-204-5p expression levels in the cochleae of aged C57BL/6 mice increased, whereas SIRT1 expression levels decreased, and miR-204-5p and SIRT1 expression levels were negatively correlated. ERS and increased 8-OHDG levels were observed in aged C57BL/6 mice. In a model of inner ear cell aging, H_2_O_2_ treatment induced increases in miR-204-5p expression and ERS-mediated apoptosis. MiR-204-5p was found to target SIRT1 and inhibit its expression. SIRT1 activation and a miR-204-5p inhibitor promoted HEI-OC1 cell proliferation and reduced apoptosis. The miR-204-5p inhibitor regulated expression of the ERS proteins PERK, ATF4, and CHOP to upregulate Bcl-2 and downregulate Bax.

**Conclusion:**

This study identified the roles of miR-204-5p and SIRT1 in deafness in C57BL/6 mice and investigated the loss of cochlear outer hair cells and the involvement of apoptosis and ERS in deafness.

## 1. Introduction

Hearing loss (HL), which is characterized by degenerative changes in the auditory organs, is progressive and affects high-frequency hearing [1]. Genetic and environmental factors are the main causes of age-related deafness [2, 3]. As the population ages, senile deafness has become an increasingly prominent and important social issue [4], as senile deafness not only negatively affects the social activity of elderly people but also has a serious effect on the quality of life on these individuals and imposes a heavy burden on individuals with senile deafness, their families, and society [5, 6]. Scientific exploration of the factors involved in the development of hearing impairment can facilitate clinical practice in related fields and provide more effective support and guidance for hearing health care.

Mammalian outer hair cells (OHCs) are long and cylindrical, and acoustic stimulation around the long axis of OHCs results in the elongation and shortening of OHCs [7, 8]. Hair cell stereocilia are at the core of electromechanical transduction, in which sound vibration is converted into a neural signal that can be interpreted by the brain [9, 10]. HEI-OC1 cells are a conditionally immortalized cell line derived from the mouse organ of Corti and express specific markers of inner ear cells [11]. HEI-OC1 cells are used to study the cellular and molecular mechanisms of ototoxicity and to screen new drugs for potential ototoxic or otoprotective properties [12, 13]. C57BL/6J mice are the most widely used inbred mouse line and the first inbred mouse line whose whole genome has been sequenced. Although this strain is tolerant of a variety of tumors, C57BL/6J mice are widely used as a genetic background strain for the expression of mutated genes [14]. This strain is homozygous for Cdh23^ahl^ (the cadherin 23 gene), an age-related HL mutation that causes progressive HL after 10 months of age [15]. The pathogenesis of deafness in C57BL/6 mice is not fully understood, and strategies for the prevention and treatment of deafness are lacking.

Recent studies have shown that miRNAs are abundantly expressed in various cell types in the animal cochlea [16], and miR-204-5p has been reported to protect HEI-0C1 cochlear cells in an apoptosis-dependent manner [17]. MiR-204-5p is also involved in ER stress (ERS) [18]. The ER is an important organelle for protein folding and quality control in eukaryotic cells, and ER homeostasis is essential for maintaining cellular function [19]. When ERS is too strong, the balance of the ER is disrupted, and intracellular homeostasis cannot be restored, which ultimately triggers cell apoptosis [20]. Previous reports have shown that SIRT1 regulates ERS to slow aging-related pathologies [21]. ERS is involved in various diseases, such as Alzheimer’s disease and Parkinson’s disease [22]. CHOP is typically maintained at a low level in the cytoplasm, but under ERS, the ER transmembrane protein PERK can activate CHOP [23]. ATF4 is a core regulator of the ERS response pathway, an adaptive cellular response to ERS [24]. The ER can also directly activate the apoptotic pathway through ERS-mediated calcium leakage into the cytoplasm, which leads to the activation of death effectors [25]. ATF4-CHOP-mediated induction of several proapoptotic genes (such as Bax) and inhibition of the synthesis of the antiapoptotic protein Bcl-2 also contribute to apoptotic cell death [25]. These findings suggest that ERS-induced apoptosis may be involved in the progression of several HL disorders.

Few studies have investigated the effects of miR-204-5p, which targets SIRT1, on ERS in the injured cochlear inner ear cell line HEI-OC1 in C57BL/6 mice. Therefore, the present study determined the expression of miR-204-5p and SIRT1 in young *versus* aged C57BL/6 mice and detected ERS. An in vitro model of oxidative damage was established using H_2_O_2_ in HEI-OC1 inner ear cells, and the effects of miR-204-5p on cell proliferation, apoptosis, and ERS were observed. Additionally, whether the targeting of SIRT1 by miR-204-tp to regulate ERS in inner ear cells can promote hearing recovery was investigated.

## 2. Materials and methods

### 2.1 Animals and grouping

C57BL/6 mice were housed one per cage in the same environment on a 12-hour day/night cycle with free access to food and water. The mice grew and aged normally. Twenty male C57BL/6 mice were divided into two groups containing 10 mice each: the young group (2 months old) and the aged group (15 months old). The results were recorded by a blinded observer. Cochlear tissues from the 2-month-old and 15-month-old mice were subjected to Western blotting, RT‒qPCR, hematoxylin and eosin (H&E) staining, and immunohistochemistry. All animals were injected intraperitoneally with 1% pentobarbital sodium (50 mg/kg) before cervical dislocation, after which cochlear tissue was obtained for subsequent experiments. All experimental procedures were performed following the guidelines for the use of experimental animals from Chongqing Medical University. All animal experiments complied with the U.K. Animals (Scientific Procedures) act and are reported as outlined by the ARRIVE guidelines.

### 2.2 Auditory brainstem response (ABR) recordings

The experimental mice were anesthetized with 0.75% pentobarbital sodium (0.1 mg/kg), and their auditory evoked potential (ABR) was measured using a smart auditory evoked potentiometer (Intelligent Hearing Systems, USA) with short, pure-tone stimulation at varying frequencies (8 kHz, 16 kHz, and 32 kHz) [17]. The intensity began at 80 dB and gradually decreased until the elicited wave III reached the minimum stimulation threshold, at which point the ABR threshold for each mouse was determined.

### 2.3 RNA extraction and real-time quantitative PCR

Total RNA was isolated from tissues or cells using TRIzol (Invitrogen, USA), and the RNA concentration was measured. Five hundred nanograms of RNA was reverse transcribed. The resulting cDNA was diluted and used as a template for real-time quantitative PCR on an ABI7500 (Applied Biosystems) PCR instrument with the following program: predenaturation at 94°C for 2 min, followed by 40 cycles of 94°C for 15 s, 60°C for 1 min, and 72°C for 10 min. Relative miRNA expression was calculated via the 2^-ΔΔCt^ method. The following primer sequences were used: miR-204-5p (forward primer): 5′-CCAGATCTGGAAGAAGATGGT-3′ and (reverse primer): 5′-GCGAATTCACAGTTGCCTACA-3′; U6 (forward primer): 5′-CTCGCTTCGGCAGCACA-3 and (reverse primer): 5′-AACGCTTCACGAATTTGCGT-3′.

### 2.4 Immunohistochemistry

Paraffin sections were routinely dewaxed and hydrated and then blocked with 10% goat serum (AR1009, Bositer, China), after which diluted antibodies were added dropwise and incubated overnight at 4°C. Biotin-labeled secondary antibodies (1:100, Servicebio, GB23303, China) were added dropwise, after which horseradish peroxidase (HRP)-labeled streptavidin and 3,3’-diaminobenzidine (DAB) were added for color development. primary antibodies against the following were used: CHOP (1:100, PA5-104528, Thermo Fisher, USA) and 8-OHDG (1:100, Bioss, bs-1278R, China). Image**-**Pro Plus 6.0 analytical software was used for semiquantitative analysis. Five sections were randomly selected from the field of view, and the positive staining area (brown) was calculated.

### 2.5 H&E staining

Mouse cochlear tissues were fixed overnight in a 4% formaldehyde solution, dehydrated in 70%, 80%, 95%, and 100% ethanol, cleared in xylene, and placed in dissolved paraffin wax for embedding. Then, the embedded tissues were sliced to a thickness of 4 μm on a microtome and mounted on a glass slide. H&E staining (C0105S, Beyotime, China) was performed according to routine procedures, and the slides were mounted with neutral gum. Histopathological changes were observed under a microscope, and images were collected.

### 2.6 Western blot analysis

A total of 100 mg of mouse cochlear tissue was weighed into a mortar and ground with liquid nitrogen, after which RIPA lysis buffer containing protease and phosphatase inhibitors was added and incubated on ice for 15 min. The tissue sample was centrifuged for 30 min (4°C, 3000 r/min), after which the supernatant was collected. To extract cellular proteins, similar to the method used to extract proteins from tissues, the cells were lysed in RIPA lysis buffer, incubated on ice for 15 min, and centrifuged for 30 min. Protein quantification and denaturation were performed according to standard methods. The samples were subjected to routine electrophoresis, after which the proteins were transferred to membranes, blocked with 5% skim milk powder at room temperature for 1.5 h, and incubated with primary antibodies against ATF4 (1:1000, ab216839, Abcam), CHOP (1:1000, 15204-1-AP, Proteintech), SIRT1 (1:1000, ab110304, Abcam), PERK (1:1000, ER64553, Huabio), p-PERK (1:1000, DF7576, affinity), Bax (1:1000, 50599-2-Ig, Proteintech), Bcl-2 (1:1000, ab182858, Abcam), and β-actin (1:1000, AC026, ABclonal) overnight at 4°C. The blots were then washed 3 times with 1× TBST and incubated with secondary antibodies (1:5000, AS014, ABclonal) for 1 h at room temperature. After the blots were washed 3 times with 1× TBST, an enhanced chemiluminescence (ECL) developer was added, and images were collected and analyzed with a gel imaging system, with β-actin used as an internal reference.

### 2.7 Luciferase reporter assay

The human renal epithelial cell line 293T was used for transfection experiments. Plasmids containing the wild-type (WT) SIRT1 3’-UTR (containing the miR-204-5p-binding site) and a mutant (Mut) SIRT1 3’-UTR (without the miR-204-5p-binding site) were inserted into the vector pGL3 (Promega, Madison, WI, USA) to obtain fluorescent reporter gene plasmids. Lipofectamine 2000 (Invitrogen; Thermo Fisher Scientific, Waltham, Massachusetts, USA) was used to transfect NC-mimic and miR-204-5p into 293T cells for 24 h. The dual-luciferase reporter assay (Promega, Madison, Wisconsin, USA) was used to detect the fluorescence intensity of each group, which was used to determine the ability of miR-204-5p to target SIRT1. All plasmids were constructed at Shanghai Shenggong Biological Co., Ltd. (Shanghai, China).

### 2.8 Cell culture and experimental conditions

The HEI-OC1 cell line was kindly provided by Professor Federico Kalinec (House Ear Institute, Los Angeles, CA, USA). HEI-OC1 cells were cultured in high-glucose DMEM containing 10% FBS at 33°C under 10% CO_2_ and saturated humidity, and the cell density was found to be 80%-90% after the cells were passaged. The culture medium was replaced with fresh complete culture medium after 24 h, and the cells were ready to be used for the experiments when they entered logarithmic growth phase. In accordance with a previous study [26], after HEI-OC1 cells were treated with 1 mM H_2_O_2_ for 1 h or 2 h, the medium was replaced with fresh medium, and the cells were cultured for an additional 24 h to establish senescent inner ear cells. In addition, 10 μM resveratrol (RSV) was used to activate SIRT1.

### 2.9 CCK-8 assay

A total of 5000 cells from each group were inoculated in 96-well plates and incubated at 33°C under 10% CO_2_. Before incubation and after 24 h of incubation, 20 μL of 5 g/L CCK-8 reagent was added to each well, and the cells were incubated at 33°C under 10% CO_2_ for 4 h. After incubation, the formed crystals were allowed to fully dissolve, and the absorbance at 450–490 nm was detected with a spectrophotometer.

### 2.10 Flow cytometry to detect apoptosis

Appropriate amounts of 1× FITC-conjugated Annexin V and a 100 μg/ml propyl iodide (PI) working solution (40302ES20, Yeasen, China) were prepared. The cells in each group were collected, washed with precooled PBS at 4°C, and centrifuged at 1000 r/min for 5 min at 4°C, after which the supernatant was discarded. A total of 1× FITC-conjugated Annexin V was added to the resuspension, and the cell density was adjusted to 1×10^6^/ml. Five microliters of Alexa Fluor 488 Annexin V and 1 μL of the 100 μg/ml PI working solution were added to 100 μL of each cell suspension, after which the cells were incubated at room temperature for 15 min in the dark. A total of 400 μL of FITC-conjugated Annexin was added, and 1×10^4^ cells were detected via flow cytometry. The data were analyzed via Cell Quest software to calculate the percentage of apoptotic cells.

### 2.11 Transfection experiments

siRNAs were synthesized by GenePharma (Shanghai, China). siRNA transfection was carried out with Heiff Trans^TM^ Liposomal Transfection Reagent (Yeasen, Shanghai, China) according to the manufacturer’s instruction. One microliter of the transfection reagent was diluted in 50 μL of serum-free medium and incubated at room temperature for 5 min. The diluted siRNA mixed with transfection reagent was incubated at room temperature for 20 min to form DNA‒liposome complexes. The liposome complexes (100 μL) were added to each well of a cell culture plate, which was then incubated at 37°C under 5% CO_2_ for 24–48 h.

### 2.12 Statistical analysis

The experimental data were statistically analyzed via SPSS 20.0 statistical software, and the experimental data are expressed as the means ± standard deviations ($\bar{X}$ ± SDs). An independent t test was used for comparisons between two groups, one-way ANOVA was used for comparisons among multiple groups, and an LSD test was used for further pairwise comparisons. P<0.05 was considered to indicate statistical significance.

## 3. Results

### 3.1 ERS and the expression of miR-204-5p and SIRT1 in young and aged mice

**Fig 1:**
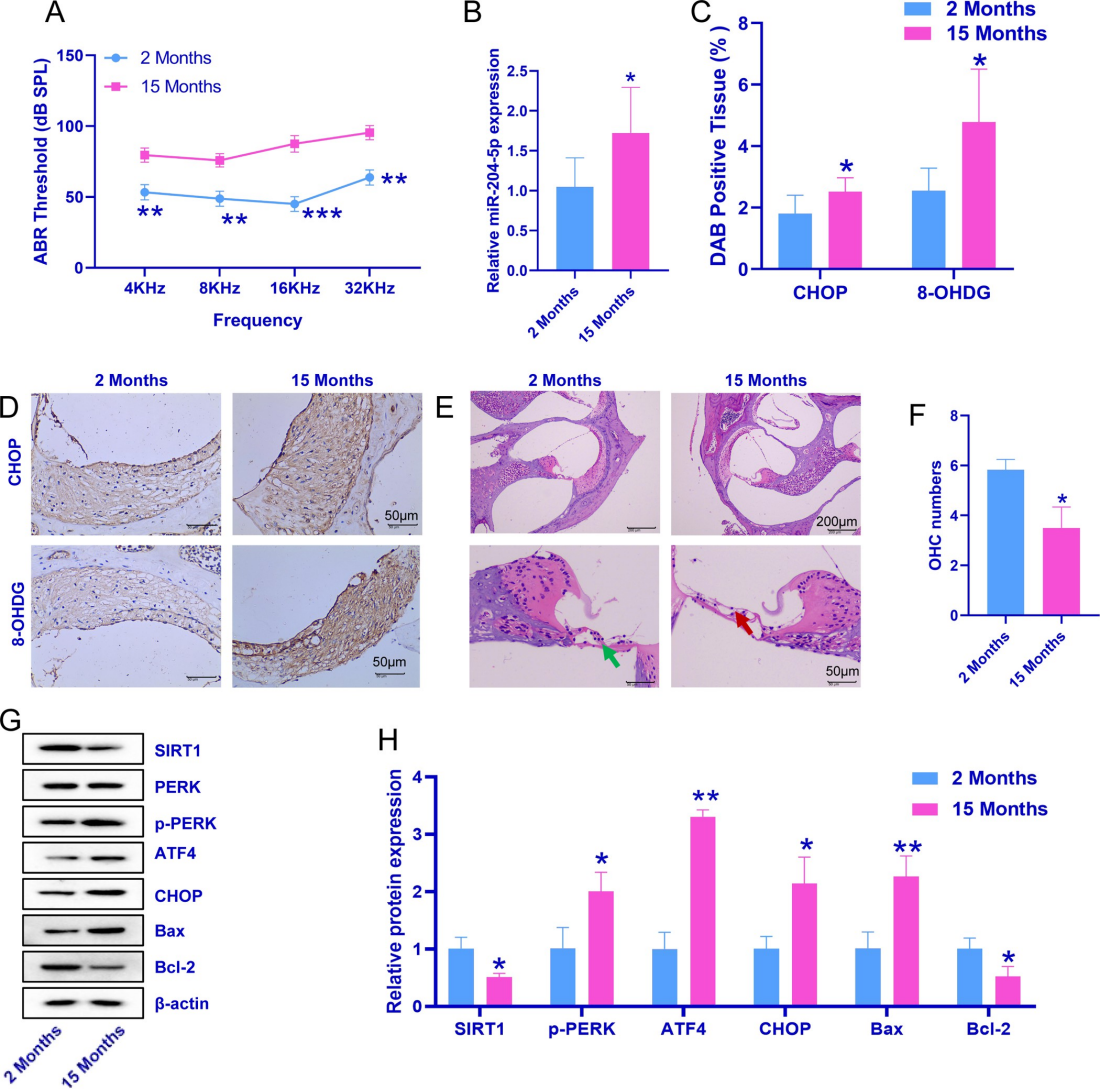
Differences in the expression of miR-204-5p and SIRT1 in inner ear cells and endoplasmic reticulum stress in the cochlea of young mice and aged mice with hearing impairment. A: Detection of ABR thresholds. B: Relative expression of miR-204-5p in 2-month-old and 15-month-old mice. C: Statistical analysis of CHOP expression and 8-OHDG levels. D: Representative images of cochlear tissue following CHOP and 8-OHDG immunohistochemistry. E: Representative images of H&E-stained sections. F: OHC numbers. G: Representative images of ATF4, CHOP, SIRT1, PERK, p-PERK, Bax, and Bcl-2 protein bands. H. Relative protein expression of ATF4, CHOP, SIRT1, p-PERK, Bax, and Bcl-2. Statistical analysis of the relative ATF4, CHOP, SIRT1, PERK, and p-PERK protein expression levels. The data are expressed as the means ± SDs. A t test was used for comparisons between two groups; *p ˂ 0.05 and **p ˂ 0.01.

ABR recordings to determine the hearing threshold of 2-month-old and 15-month-old mice were collected. As shown in Fig 1A, compared with those of the young group, the ABR thresholds of the aged group were greater at 4, 8, 16, and 32 kHz. Compared with that in 2-month-old mice, relative miR-204-5p expression was increased in the 15-month-old mice (Fig 1B). Moreover, CHOP expression and 8-OHDG levels were greater in 15-month-old mice than in 2-month-old mice (Fig 1C and 1D). In addition, the aged group had fewer OHCs than the young group, and the arrangement of these OHCs was disordered (Fig 1E and 1F). Furthermore, ATF4, CHOP, p-PERK, and Bax protein levels were greater in the aged group than in the young group, and the levels of SIRT1 and Bcl-2 were lower in the aged group (Fig 1G and 1H). The above experimental results indicated that aged C57BL/6 mice presented impaired hearing, ERS, and a disordered cochlear cell arrangement.

### 3.2 Expression of miR-204-5p and SIRT1 in an inner ear cell model with H_2_O_2_-induced senescence

**Fig 2:**
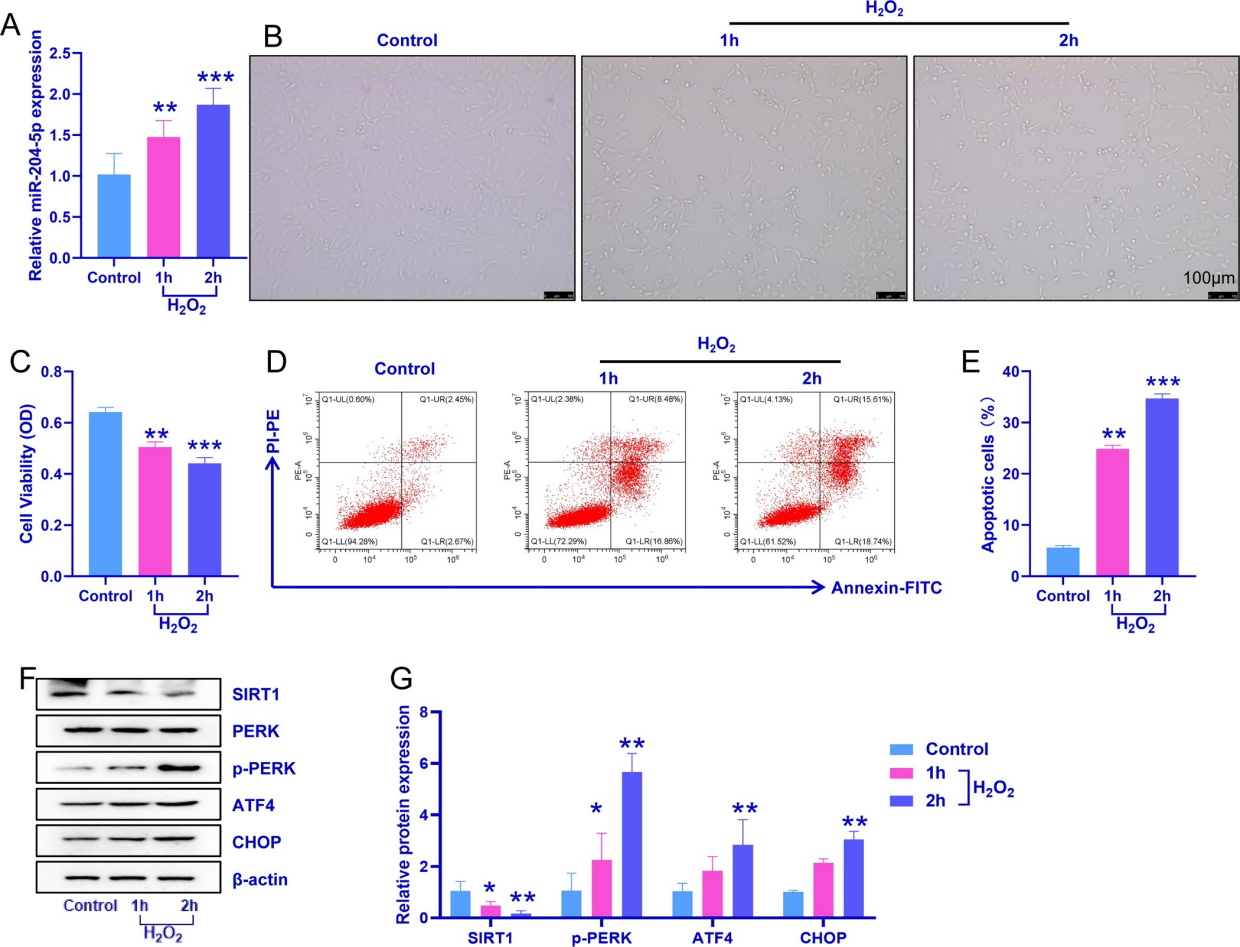
Expression of miR-204-5p and SIRT1 in a model of inner ear cells with H_2_O_2_-induced senescence. A: Effect of H_2_O_2_ treatment on miR-204-5p expression in HEI-OC1 cells. B: Imaging of HEI-OC1 cells after H_2_O_2_ treatment. C: Proliferation of HEI-OC1 cells determined by the CCK-8 assay. D: Representative images of apoptotic cells determined by flow cytometry. E: Statistical analysis of the proportion of apoptotic cells. F: Representative images of ATF4, CHOP, SIRT1, PERK, and p-PERK protein bands. G. Relative protein expression of ATF4, CHOP, SIRT1, PERK, and p-PERK. Statistical analysis of the relative protein expression levels of ATF4, CHOP, SIRT1, and p-PERK. The experiment was independently repeated three times. The data are expressed as the means ± SDs; *p ˂ 0.05, **p ˂ 0.01, and *** p ˂ 0.0001, compared with the control group.

To verify the effects of miR-204-5p and SIRT1 on hearing in vitro, we used H_2_O_2_ to establish a model of aging HEI-OC1 inner ear cells. Compared with that in the control group (H_2_O_2_-free, HEI-OC1 cells incubated with PBS as a control), the expression level of miR-204-5p increased after 1 h and 2 h of H_2_O_2_ treatment (Fig 2A). As shown in Fig 2B, compared with that of the H_2_O_2_-free group (control group), the density of HEI-OC1 cells decreased after 1 h and 2 h of H_2_O_2_ treatment, and some cells appeared wrinkled and round. A CCK-8 cell proliferation assay revealed that the proliferation capacity of HEI-OC1 cells decreased after 1 h and 2 h of treatment with H_2_O_2_ (Fig 2C). Flow cytometry to detect apoptosis showed that compared with that of the control group, the number of apoptotic cells was significantly increased after H_2_O_2_ treatment for 1 h and 2 h and peaked at 2 h (Fig 2D and 2E). Similar to the results of the animal experiments, compared with those of the control group, the expression levels of ATF4, CHOP, p-PERK, and Bax were increased, and the expression levels of SIRT1 and Bcl-2 were decreased in a senescent inner ear cell model induced by 1 h and 2 h of treatment with H_2_O_2_. The above experimental results suggest that H_2_O_2_-induced apoptosis of senescent inner ear cells occurs as a result of ERS.

### 3.3 Effect of SIRT1 on ERS in a model of H_2_O_2_-induced senescence in inner ear cells

**Fig 3:**
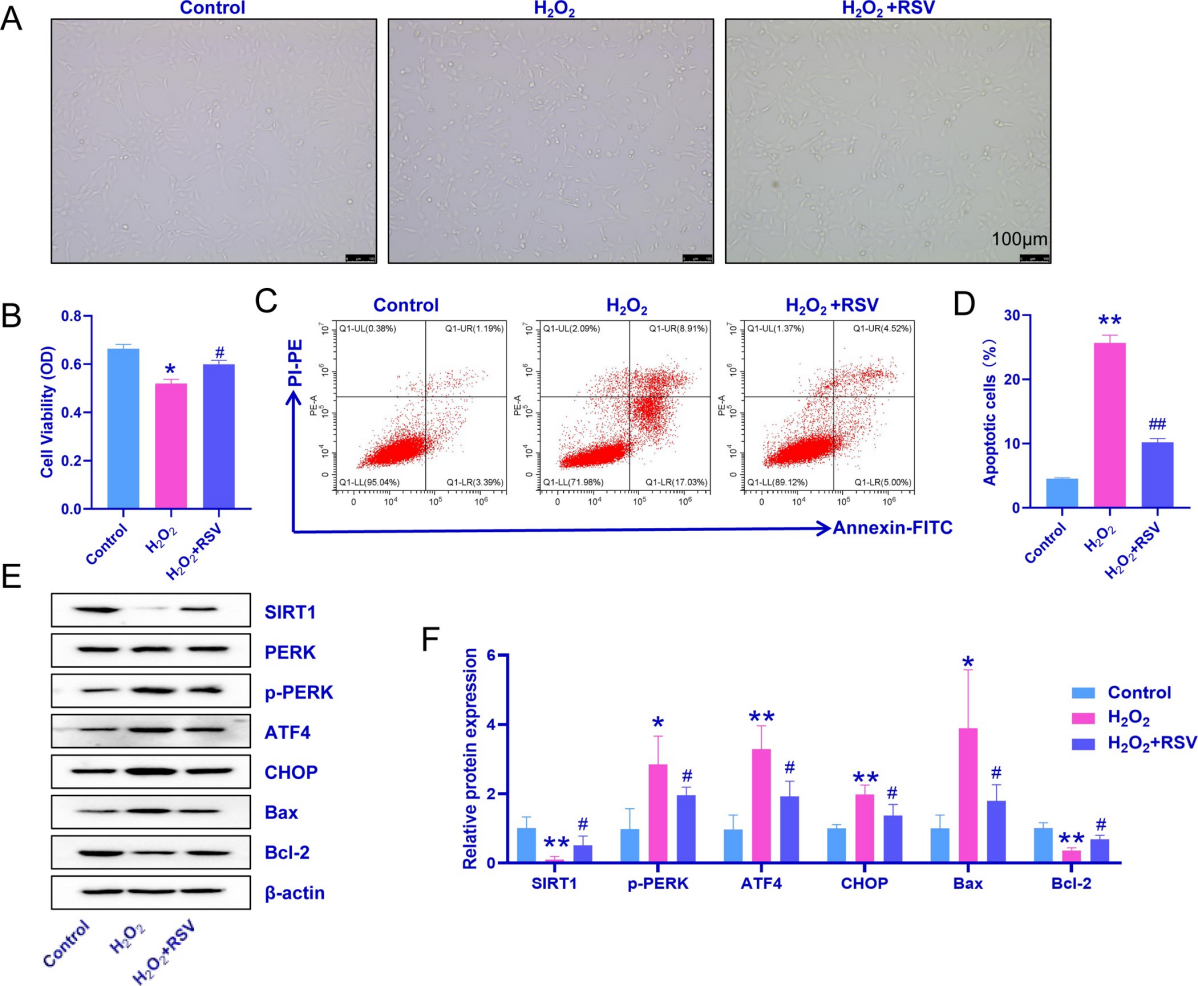
The SIRT1 agonist RSV regulates H_2_O_2_-induced senescence in inner ear cells with ERS and affects cell proliferation and apoptosis. A: Effects of the SIRT1 agonist RSV on the growth of inner ear cells with H_2_O_2_-induced senescence. B: Statistical analysis of the results of the CCK-8 proliferation assay. C: Representative images of apoptotic cells after RSV treatment. D: Results of the statistical analysis of the proportion of apoptotic cells. E: Representative images of ATF4, CHOP, SIRT1, PERK, p-PERK, Bax, and Bcl-2 protein bands. F: Relative protein expression of ATF4, CHOP, SIRT1, p-PERK, Bax, and Bcl-2. The experiment was independently repeated three times. The data are expressed as the means ± SDs, n = 10. *p ˂ 0.05, and **p ˂ 0.01 compared with the control group. #p ˂ 0.05 and ##p ˂ 0.01 compared with the H_2_O_2_ group.

Because SIRT1 expression was reduced in senescent inner ear cells, we chose the SIRT1 agonist RSV [27] to study the effects of SIRT1 and ERS on apoptosis. As shown in Fig 3A, compared with that in the H_2_O_2_ group, the number of shrunken and round cells was reduced after RSV treatment. A CCK-8 cell proliferation assay revealed that the reduction in cell proliferation caused by H_2_O_2_ was reversed after RSV treatment (Fig 3B). In addition, flow cytometry revealed that H_2_O_2_-induced apoptosis was effectively reduced by RSV (Fig 3C and 3D). Previous experiments revealed changes in the expression of ATF4, CHOP, SIRT1, PERK, p-PERK, Bax, and Bcl-2 after H_2_O_2_ treatment. Compared with those following H_2_O_2_ treatment, RSV treatment downregulated ATF4, CHOP, p-PERK, and Bax expression and upregulated SIRT1 and Bcl-2 expression. These experimental results demonstrated the involvement of SIRT1 in apoptosis due to H_2_O_2_-induced ERS in aging inner ear cells.

### 3.4 Bioinformatics analysis of SIRT1-binding sites in miR-204-5p

**Fig 4:**
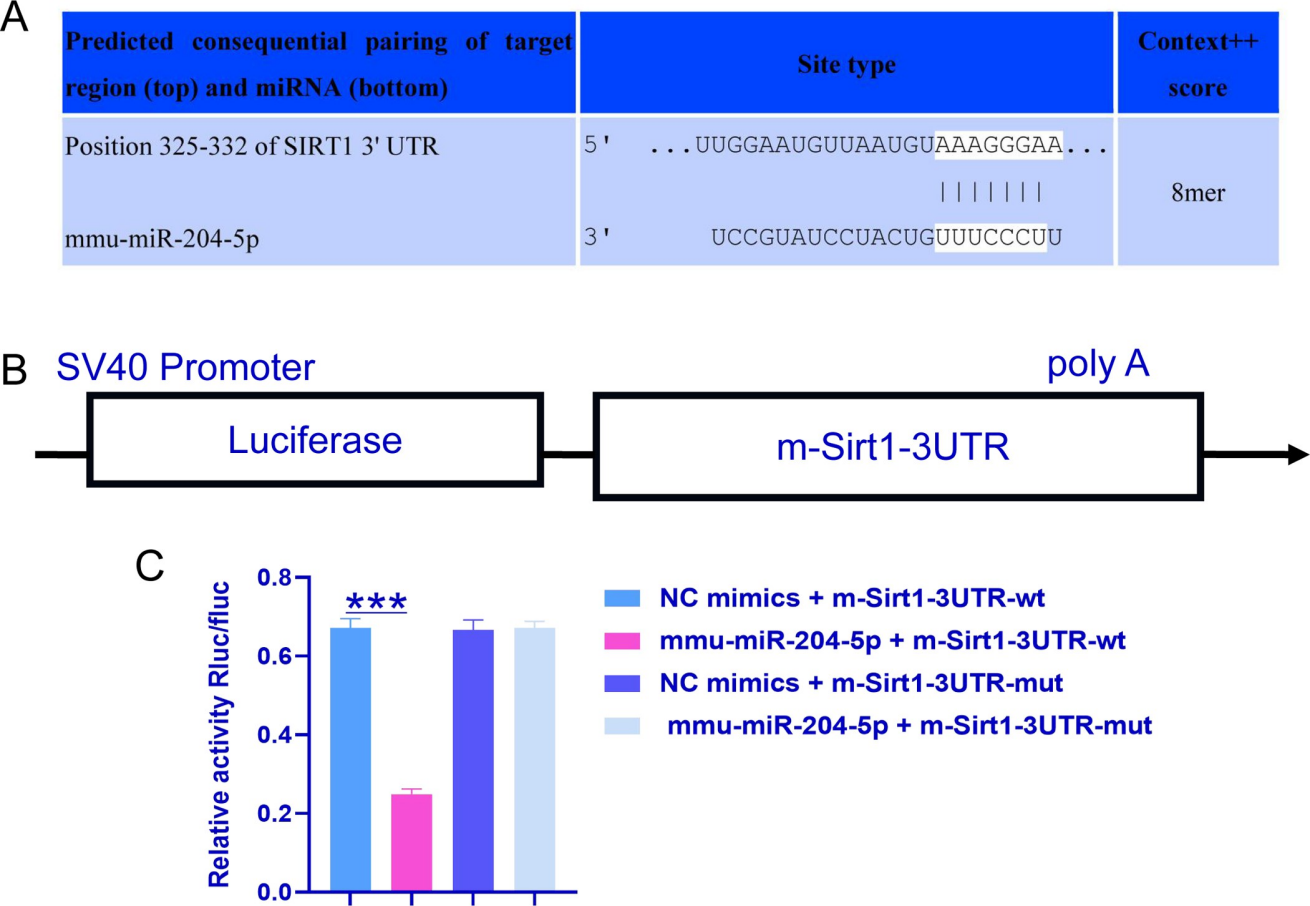
Dual-luciferase reporter gene assay to determine whether miR-204-5p interacts with the Sirt1 3’UTR in 293T cells. A: TargetScan software predicted base pairing between the 3′UTR of SIRT1 mRNA and the sequence of miR-204-5p. B: The 3’UTR of SIRT1 was cloned into a luciferase reporter vector to generate a SIRT1-3’UTR reporter plasmid. C: Relative luciferase activity of 293T cells transfected with the NC mimic or miR-204-5p mimic and the WT or Mut SIRT1 3’UTR reporter plasmid. The experiment was independently repeated three times. The data are expressed as the means ± SDs; *** p ˂ 0.0001.

Experiments in both animals and cells revealed that the levels of miR-204-5p and SIRT1 were altered in aging inner ear cells. Since miR-204-5p functions by negatively regulating the expression of target genes, TargetScan (Whitehead Institute for Biomedical Research, Cambridge, MA, USA) was used to predict the targets of miR-204-5p. As shown in Fig 3A, perfect base pairing was observed between the 3’UTR of SIRT1 mRNA and the seed sequence of miR-204-5p. A SIRT1 luciferase reporter gene was selected to construct a plasmid used to validate the relationship between miR-204-5p and SIRT1 (Fig 4B). Compared with the NC mimic (a miRNA negative control to eliminate nonspecific effects), the miR-204-5p mimic significantly inhibited the transcriptional activity of the SIRT1 3’UTR-wt plasmid, whereas neither the NC mimic nor the miR-204-5p mimic affects the transcriptional activity of the SIRT1 3’UTR-mut plasmid (Fig 4C). These experimental results confirmed that SIRT1 is a target of miR-204-5p.

### 3.5 Effect of miR-204-5p-mediated targeting of SIRT1 on apoptosis due to H_2_O_2_-induced ERS in senescent inner ear cells

**Fig 5:**
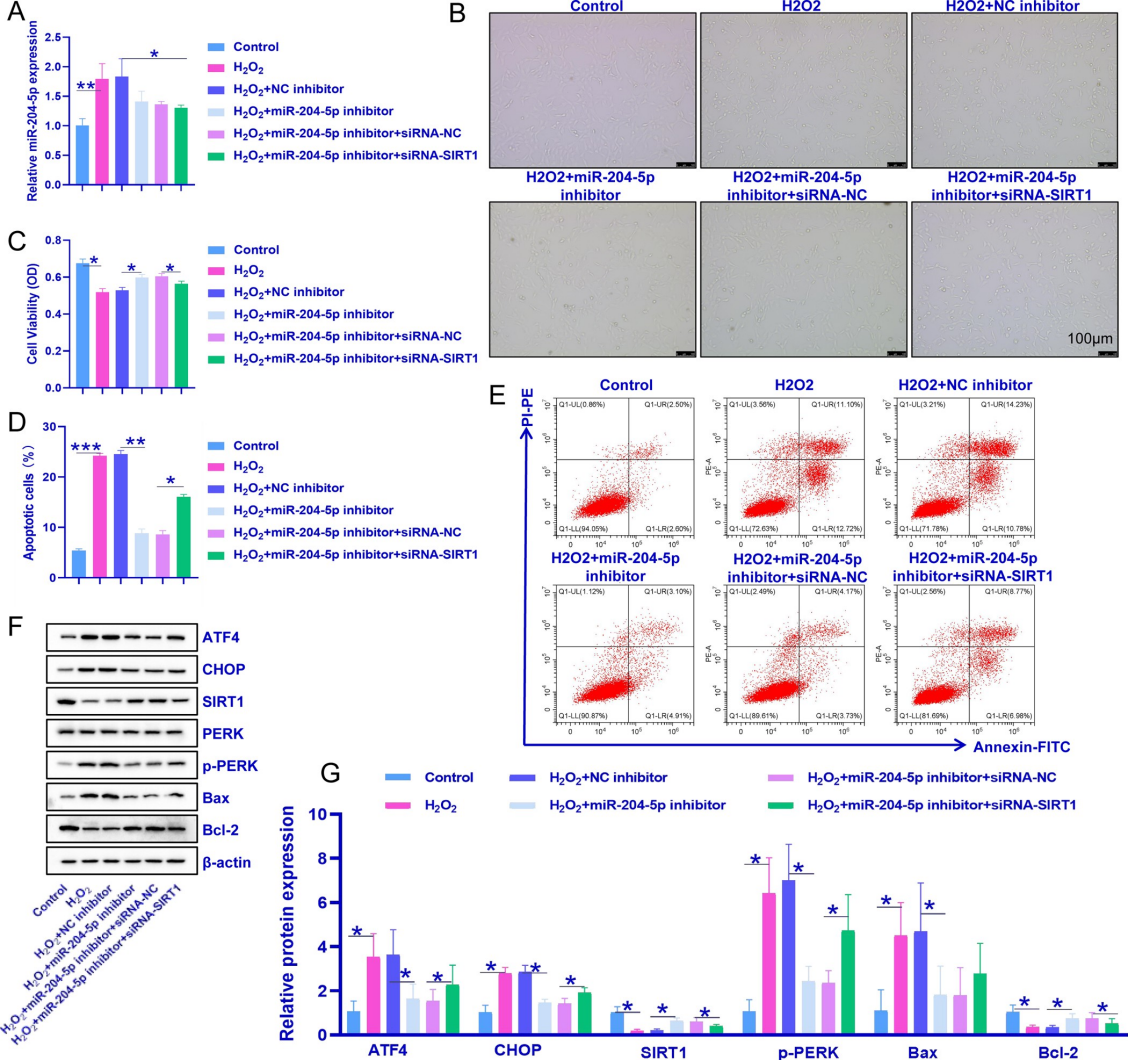
miR-204-5p targets SIRT1 to affect H_2_O_2_-induced ERS in aging inner ear cells and thereby affects cell proliferation and apoptosis. A: Effect of a miR-204-5p inhibitor on the expression of miR-204-5p in HEI-OC1 cells. B: Imaging of HEI-OC1 cells treated with the miR-204-5p inhibitor and siRNA-SIRT1. C: Statistical analysis of the effects of the miR-204-5p inhibitor and siRNA-SIRT1 on the proliferation of HEI-OC1 cells, as determined by the CCK-8 assay. D: Statistical analysis of the effects of the miR-204-5p inhibitor and siRNA-SIRT1 on apoptosis. E: Representative images of apoptotic cells. F: Representative images of ATF4, CHOP, SIRT1, PERK, p-PERK, Bax, and Bcl-2 protein bands. G: Relative protein expression of ATF4, CHOP, SIRT1, p-PERK, Bax, and Bcl-2. The experiment was independently repeated three times. The data are expressed as the means ± SDs; *p ˂ 0.05, **p ˂ 0.01, and ***p ˂ 0.001.

After the luciferase reporter gene assay was used to verify the ability of miR-204-5p to target SIRT1, we used a combination of a miR-204-5p inhibitor, a miR-204-5p inhibitor, and siRNA-SIRT1 (a siRNA used to interfere with SIRT1 expression) to verify that miR-204-5p deactivates ERS-induced apoptosis via SIRT1. As shown in Fig 1A, H_2_O_2_ increased miR-204-5p expression in senescent inner ear cells; the miR-204-5p inhibitor decreased miR-204-5p expression compared with that in the H_2_O_2_ group, and the siRNA had no effect. Compared with that in the H_2_O_2_ group, the miR-204-5p inhibitor promoted HEI-OC1 cell growth, and this effect was diminished when siRNA-SIRT1 was used to silence SIRT1 (Fig 5B and 5C). In addition, the number of apoptotic cells was lower in the miR-204-5p inhibitor group than in the H_2_O_2_ group and greater in the siRNA-SIRT1 group than in the H_2_O_2_+miR-204-5p inhibitor group (Fig 5D and 5E). Compared with those in the H_2_O_2_ group, the protein levels of ATF4, CHOP, p-PERK, and Bax were downregulated, and the protein levels of SIRT1 and Bcl-2 were upregulated after treatment with the miR-204-5p inhibitor. In contrast, the effect of the miR-204-5p inhibitor was inhibited by siRNA-SIRT1 (Fig 5F and 5G). Moreover, the NC inhibitor and siRNA-NC did not affect cell proliferation, apoptosis, or the expression of the proteins involved. In conclusion, the effect of miR-204-5p on senescent inner ear cells involves SIRT1.

## 4. Discussion

HL causes a decline in quality of life for approximately 30% of older people and is becoming a growing global public health concern [1]. The use of cochlear implants in the treatment of HL has shown promise, but a consensus on the ethics involved in the use of cochlear implants need to be reached. In addition, the high cost of cochlear implants and the risks of surgery make it difficult to apply cochlear implants in most cases [28]. HL often has no obvious symptoms in the early stage, so HL is often at a later stage when it is discovered. For HL patients who have progressed to moderately severe HL or worse with a long onset, irreversible degenerative changes have occurred in the auditory organs, and the use of traditional diagnostic and therapeutic methods is limited. Therefore, the study of miRNAs involved in HL and their mechanisms provides potential targets for miRNA-based HL treatments and opens new avenues for the prevention and treatment of HL.

In this study, 2-month-old and 15-month-old C57BL/6 mice were selected to mimic the clinical status of young and elderly people. ABR testing [29] can be used to identify HL in aged rats, and this animal model can mimic the clinical signs of HL in old age. This study revealed the increased levels of CHOP and 8-OHDG in the cochlear tissue of aged C57BL/6 mice. 8-OHDG is a marker of oxidative damage [30], and its presence implied that damage to the cochlear tissue occurred in the aged mice. CHOP is involved in ERS-mediated apoptosis [31], and its presence also implied the occurrence of ERS in aged mouse cochlear tissue. Interestingly, we also reported high miR-204-5p expression and decreased SIRT1 expression in aged mice. Weston et al. reported that miRNAs are involved primarily in genetic programs inherent in the development and function of the mammalian inner ear and that specific miRNAs can influence the formation of the sensory epithelium derived from the primitive ear neuroepithelium [32]. Previous studies revealed that miR-204 is among the factors related to proliferation and differentiation in the organ of Corti during HL [33]. Therefore, combining all the above results, we hypothesized that miR-204-5p expression in the cochlea increases with age and that excess miR-204-5p inhibits SIRT1, which leads to ERS and the apoptosis of cochlear cells, causing HL in aged C57BL/6 mice.

miRNAs are highly conserved endogenous noncoding small RNAs that negatively regulate the expression of target genes by suppressing mRNA transcription; thus, miRNAs participate in the regulation of cell growth, development, signaling, proliferation, differentiation, and apoptosis and other processes [34]. miRNAs are abundantly expressed in the cochlear tissue and regulate the growth and development of inner ear cells and apoptosis [35]. The miR-183 family plays an important role in the regulation of hair cell development in the inner ear [36]. miR-34a causes HL by regulating cortical neuronal apoptosis, which is involved in normal aging [37]. In addition, Lisheng Xie [17] reported that the miR-204-5p/Bcl-2 axis regulates cochlear apoptosis. In this study, we found that miR-204-5p expression was increased in both aged C57BL/6 mouse cochleae and inner ear cells with H_2_O_2_-induced senescence, suggesting that miR-204-5p is detrimental to the cochlea. In addition, Lihua Zhang [38] reported that miR-204 can downregulate SIRT1 expression in gastric cancer cells and reverse the SIRT1-induced epithelial‒mesenchymal transformation of gastric cancer cells. Moreover, we identified the site of miR-204-5p that binds SIRT1 via TargetScan, and a dual-fluorescence reporter gene assay confirmed the inhibitory effect of miR-204-5p on SIRT1.

In recent years, SIRT1 has become a research focus in the study of geriatric diseases. During the aging process, the central nerve, the organ of Corti at the base of the cochlea, and the cochlear nerve, which innervates the basilar membrane, atrophy, leading to the development of HL [39]. Numerous studies have confirmed that SIRT1 plays an important role in slowing the development of neurodegenerative diseases and that SIRT1 is closely related to diabetes and cardiovascular diseases; thus, SIRT1 is also known as a longevity gene [40, 41]. The expression of SIRT1 in the inner ear cells of aged C57BL/6 mice was significantly downregulated, and the expression of SIRT1 was found to positively regulate apoptosis in HEI-OC1 cells, indicating that SIRT1 is related to the onset of presbycusis [42]. The decrease in the expression of SIRT1 in response to H_2_O_2_ was accompanied by the decreased proliferation and increased apoptosis of HEI-OC1 cells. In addition, the number of apoptotic cells was reduced by treatment with the SIRT1 agonist RSV. High miR-204-5p expression was accompanied by low SIRT1 expression in HEI-OC1 cells, and animal experiments also suggested that miR-204-5p has a targeted inhibitory effect on SIRT1.

Both miR-204-5p and SIRT1 are strongly associated with ERS. In human beta cells, the miR-204-5p family is a key hub involved in the regulation of cytokine-induced ERS and proapoptotic pathways in human cells [18]. SIRT1 protects the heart from ERS-induced injury by promoting eef2k/eef2-dependent autophagy [43]. ERS is involved in the development of age-related diseases [22]. Fujinami [44] generated an animal model of cochlear cell injury induced by ERS. In the current study, ERS was observed in both aged C57BL/6 mice and H_2_O_2_-treated aged inner ear cells. The CHOP pathway is the predominant pathway by which ERS mediates apoptosis [45]. The CHOP gene promoter contains an ATF4-binding site, and the PERK-ATF4-CHOP signaling pathway increases CHOP expression when ERS-mediated apoptosis occurs [46, 47]. While we identified PERK‒ATF4‒CHOP signaling in aged C57BL/6 mice and senescent model cells, we also found that SIRT1 activation blocked this signaling pathway and that miR-204-5p inhibitors effectively activated this signaling pathway. A previous study revealed that inner ear cell apoptosis is closely related to age, and in the cochleae of aged animals, the expression of Bcl-2 is decreased, and the expression of Bax is increased; these changes in the expression of Bcl-2 and Bax are positively correlated with cochlear dysfunction [48]. In this study, ERS also resulted in decreased Bcl-2 expression and increased Bax expression.

In summary, miR-204-5p can target SIRT1 to regulate the PERK-ATF4-CHOP signaling pathway and regulate ERS. Furthermore, the inhibition of miR-204-5p can reduce apoptosis and protect HEI-OC1 cells. Our future research will aim to better understand the mechanisms of inner ear cell proliferation and apoptosis and changes in these process that occur naturally. Addressing these issues will also contribute to a better understanding of how these processes accelerate deafness in C57BL/6 mice.

## Supporting information

S1 File (XLS)

S1 Raw images (PDF)
