# Supplementary figures and images for: MiR-204-5p regulates SIRT1 to promote the endoplasmic reticulum stress-induced apoptosis of inner ear cells in C57BL/6 mice with hearing loss
Source: PLoS One. 2024 Nov 12;19(11):e0309892. doi: 10.1371/journal.pone.0309892 (PMC11556682; doi:10.1371/journal.pone.0309892)

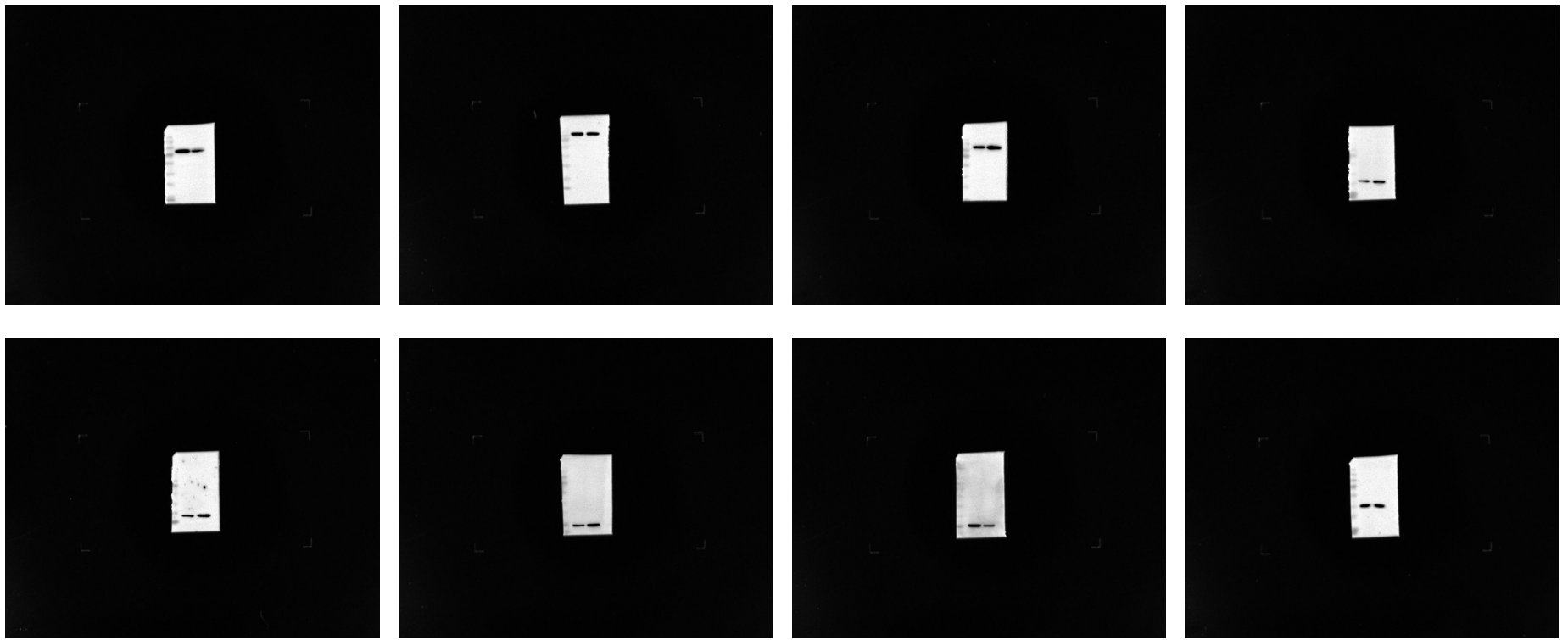

Figure 1

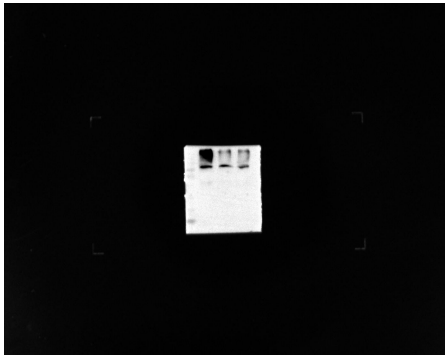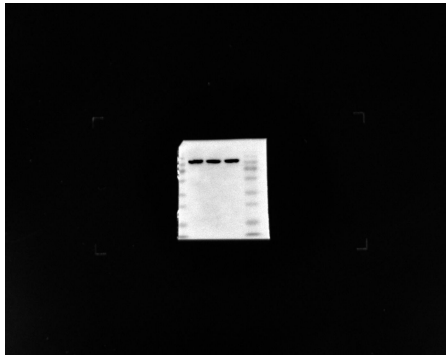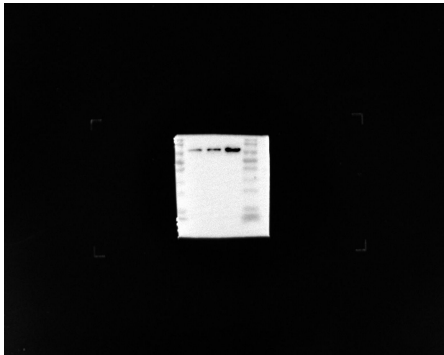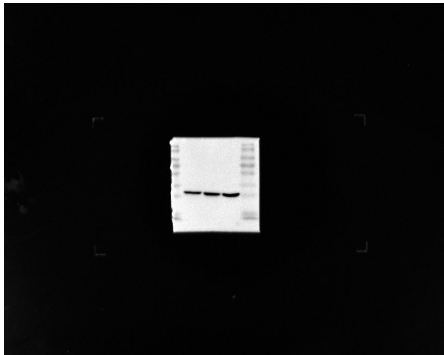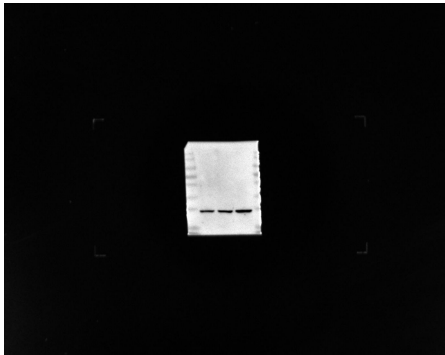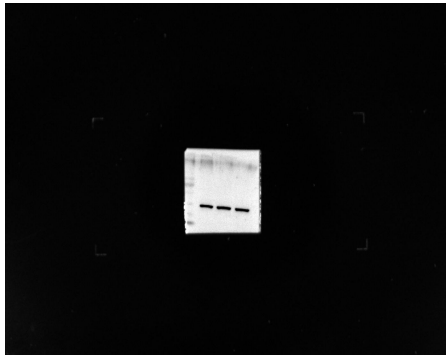

Figure 2

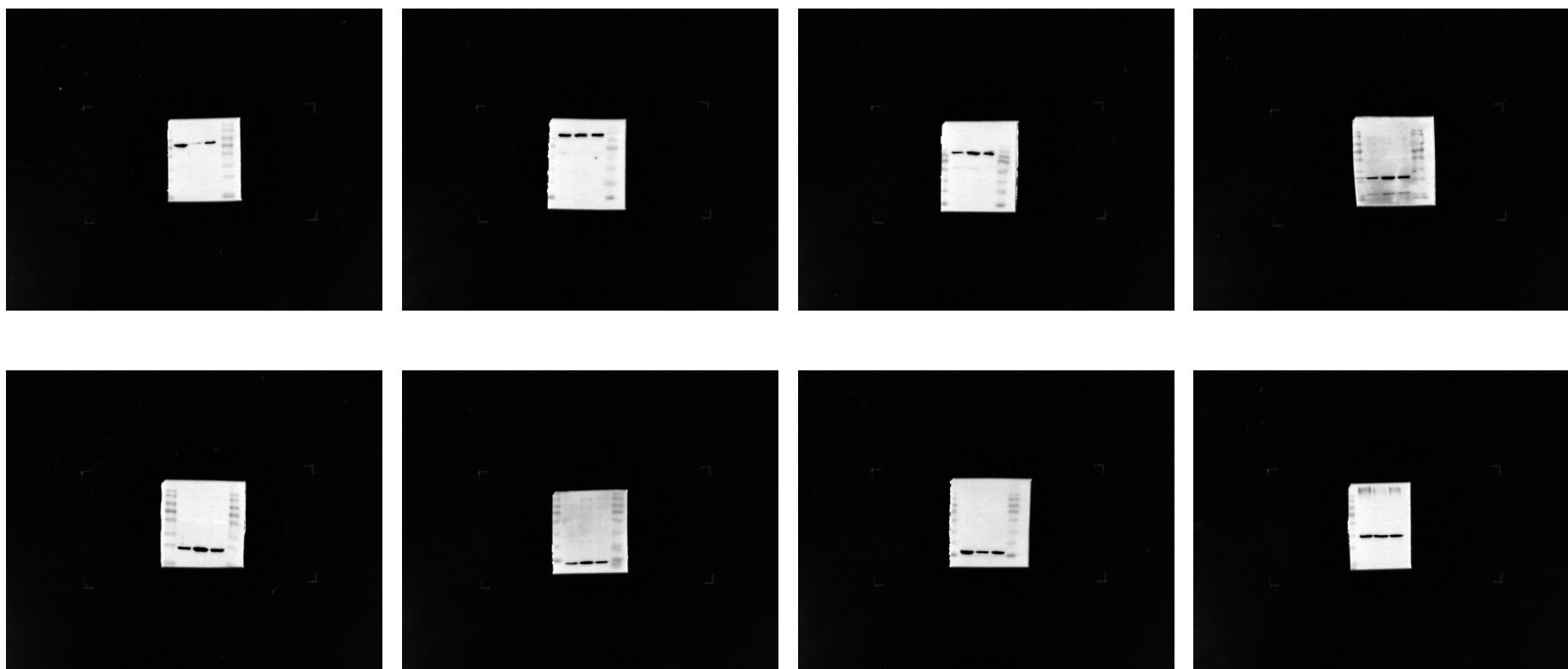

Figure 3

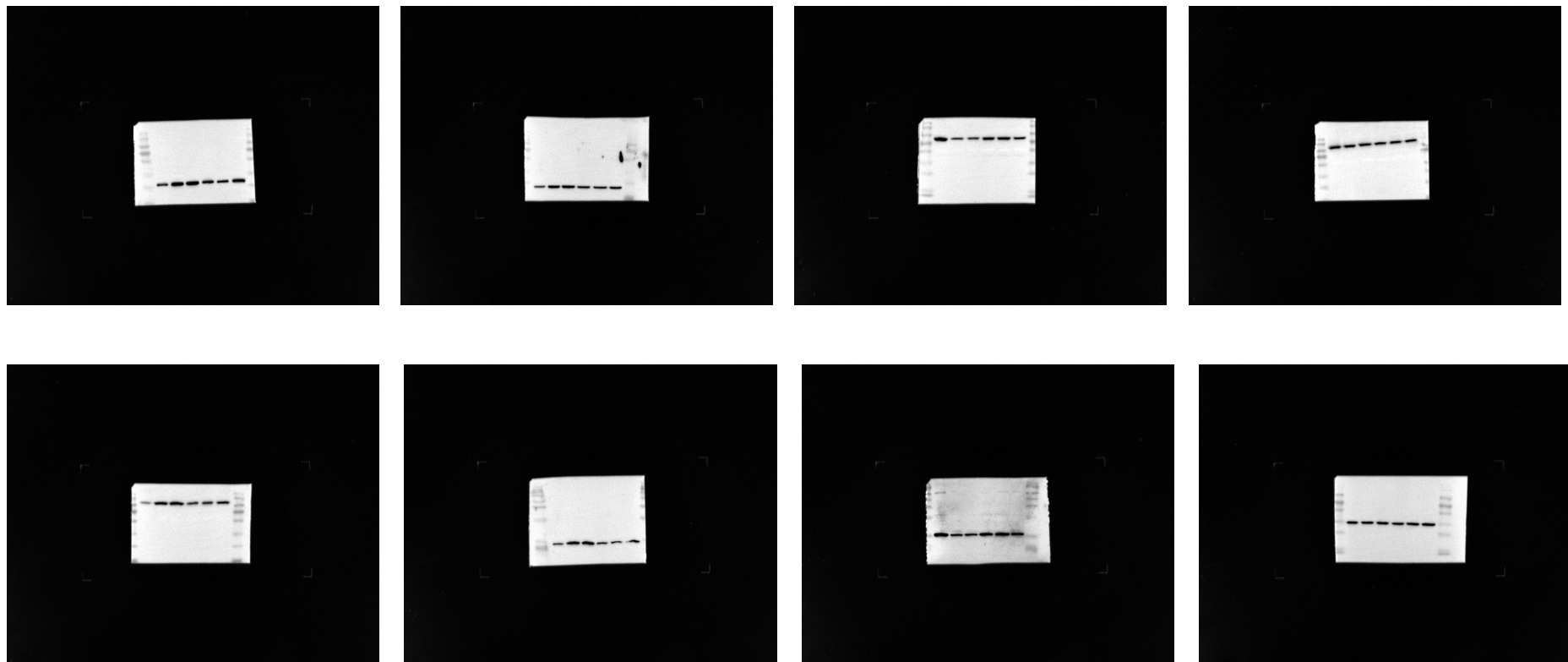

Figure 5

Supplement: S1 Raw images — (PDF) [file pone.0309892.s002.pdf]
